# Crosstalk between heterogeneous cancer-associated fibroblast subpopulations and the immune system in breast cancer: key players and promising therapeutic targets

**DOI:** 10.1186/s13046-025-03527-z

**Published:** 2025-09-01

**Authors:** Lu Liu, Weijun Wan, Yilin Chang, Luoquan Ao, Yan Xu, Xiang Xu

**Affiliations:** 1 State Key Laboratory of Trauma and Chemical Poisoning, Department of Stem Cell and Regenerative Medicine, Daping Hospital, Army Medical University, Chongqing, 400010 China; 2 Department of Breast and Thyroid Surgery, Daping Hospital, Army Medical University, Chongqing, 400010 China

**Keywords:** Cancer-associated fibroblasts, Heterogeneity, Breast cancer, Tumor microenvironment, Therapeutic strategies

## Abstract

The tumor microenvironment (TME) of breast cancer is a complex ecosystem, in which cancer-associated fibroblasts (CAFs), as the most abundant stromal cell type, meticulously construct an ecological niche that supports tumor growth through mechanisms including extracellular matrix (ECM) remodeling, secretion of bioactive factors, and interactions with neighboring cells. High-resolution technologies, including single-cell sequencing and spatial transcriptomics, have revealed the high heterogeneity, functional diversity, and spatial distribution within the CAF population. Significant differences exist in the interactions between distinct CAF subpopulations and immune cells. Through complex crosstalk with the immune system, they collaboratively establish an immunosuppressive network, becoming a core driving force for tumor immune escape. This review focuses on the latest research advances in heterogeneous subpopulations of CAFs within the breast cancer microenvironment, delves into how the complex bidirectional crosstalk between different CAF subpopulations and immune cells collaboratively shapes the tumor immune microenvironment (TIME), and summarizes various CAF-based therapeutic strategies for breast cancer, aiming to provide critical theoretical basis and novel therapeutic perspectives for the clinical translation of CAF heterogeneity research.

## Background

Breast cancer is a highly heterogeneous tumor, which is not only reflected in the molecular subtyping differences of the tumor itself, but also closely related to the complex regulatory mechanisms of the tumor microenvironment (TME) involved in its occurrence and development [1]. The TME is a dynamic ecosystem composed of various components including tumor cells, stromal cells, immune cells, and extracellular matrix (ECM), with each component synergistically driving tumor progression through a complex interaction network [2]. Among the numerous stromal cells in the TME, cancer-associated fibroblasts (CAFs) are the most abundant [2]. These activated fibroblasts have been extensively studied in recent years and are increasingly recognized as core hubs in the TME regulatory network, driving tumor progression, treatment resistance, and immune escape [3]. CAFs not only dominate ECM remodeling, but also profoundly influence the malignant behaviors of tumor cells by secreting various cytokines, growth factors and chemokines, and contacting neighboring cells directly, thus meticulously constructing a “supportive niche” for tumor progression [4].

With the application of high-resolution technologies such as single-cell sequencing and spatial transcriptomics, our understanding of CAFs is undergoing a profound transformation. These technologies have revealed a high degree of heterogeneity within the CAF population, far exceeding the scope that can be defined based on a few surface markers. Studies have not only identified functionally distinct CAF subpopulations, but also further meticulously linked these subpopulations to their specific spatial distributions within the TME, neighboring cell types (especially immune cells) and related biological functional modules by leveraging spatial dimension information [5], mapping the identity landscape, functional states, and dynamic evolutionary trajectory of CAFs in shaping the TME with unprecedented resolution.

The functional heterogeneity of CAFs is prominently reflected in their complex interactions with the tumor immune microenvironment (TIME) [6]. Substantial evidence indicates that specific CAF subpopulations are key regulators in shaping an immunosuppressive TIME. These “pro-tumorigenic” CAF subpopulations can conduct complex bidirectional crosstalk with key immune cells such as T cells, macrophages, and myeloid-derived suppressor cells (MDSCs) through diverse mechanisms, collectively creating a “cold” tumor microenvironment unfavorable for anti-tumor immune responses [7–10]. Therefore, a deep understanding of the intricate immune regulatory network mediated by different CAF subpopulations is crucial for elucidating tumor immune escape mechanisms and breaking through the current bottlenecks in immunotherapy.

Taken together, all these features highlight the importance of CAFs. This article systematically reviews the heterogeneous CAF subpopulations in the breast cancer TME, focusing on analyzing the dynamic interaction mechanisms between different CAF subpopulations and various immune cells. These latest knowledge not only delineates a comprehensive landscape of CAFs in breast cancer, but also promotes the proposal of new precision therapy strategies for breast cancer.

## CAFs in breast cancer microenvironment

As a core stromal cell type in the breast cancer TME, CAFs exhibit high heterogeneity, which is reflected in many aspects including origin, markers, function, phenotype, and molecular characteristics. In recent years, single-cell RNA sequencing (scRNA-seq), spatial transcriptomics technologies, and spatial proteomics have developed rapidly. These technologies have revealed the characteristics of CAF subpopulations and their spatial distribution within the TME at high resolution, providing important tools for understanding CAF heterogeneity.

### Origins of CAFs

In breast cancer, the origins of CAFs are diverse and complex, involving multiple cell types and molecular mechanisms. Studies have shown that CAFs primarily originate from resident fibroblasts in breast tissue. These cells are activated by factors such as TGF-β [11–14] and SDF-1 [11, 15] secreted by tumor cells, transforming into CAFs with pro-tumorigenic properties via the TGF-β/Smad or the JAK/STAT3 signaling pathway [16, 17]. Notably, the maintenance of CAF activation appears to be dependent on the continued presence of stimulation. Recent evidence suggests that withdrawal of TGF-β can lead to the partial deactivation of CAFs or their complete reversion to a quiescent state. Conversely, prolonged exposure to activating stimuli may stabilize their activated phenotype, making reversal more difficult to achieve [18]. This suggests that the timing and duration of stimulation are key factors in determining the stability of the CAF activation phenotype. In addition, bone marrow mesenchymal stem cells (BMSCs) are recruited to the TME and induced to differentiate into CAFs by tumor-derived osteopontin (OPN) [19, 20] or basic fibroblast growth factor (bFGF) [21], promoting tumor growth and metastasis. Adipose-derived stem cells (ASCs) in breast cancer are converted into CAFs via the Wnt/β-catenin [22] or the TGF-β/Smad signaling pathway [23], which is particularly significant in obese patients [24]. These activation pathways are not unique to breast cancer. Similar mechanisms also drive the induction of CAFs in other cancers, such as pancreatic cancer [25], colorectal cancer [26], and lung cancer [27], indicating that these regulatory networks are conserved across tumor types. Cancer stem cells (CSCs) and pericytes may also serve as sources of CAFs in the breast cancer microenvironment [28, 29]. Epithelial cells and endothelial cells differentiate into CAFs via epithelial-mesenchymal transition (EMT) and endothelial-mesenchymal transition (EndMT), respectively, supporting angiogenesis and tumor invasion [30–32]. Importantly, the primary origin of CAFs varies across tumor types. For instance, pancreatic stellate cells are the main precursors of CAFs in pancreatic cancer, while CAFs in liver cancer often originate from hepatic stellate cells [10]. This diversity of origins, shaped by tissue-specific microenvironments, lays the foundation for the functional and phenotypic heterogeneity of CAFs.

### Markers and functional phenotypes of CAFs

Early studies primarily identified CAFs based on the expression of proteins such as α-smooth muscle actin (α-SMA), fibroblast activation protein (FAP), fibroblast specific protein 1 (FSP1/S100A4), platelet-derived growth factor receptor (PDGFR) α/β, and caveolin-1 (Cav-1) [33]. However, the expression of these markers varies significantly among different CAF populations, making it difficult to fully define the subtypes and functions of CAFs [34]. Notably, the relationship between the expression levels of these markers and clinical outcomes is complex and contradictory. For instance, the high expression of the classic CAF marker FAP in ductal carcinoma in situ (DCIS) indicates strong tumor invasiveness and poor prognosis [35], whereas in invasive ductal carcinoma (IDC), it is significantly correlated with longer overall survival (OS) and disease-free survival (DFS) [36]. α-SMA, as a marker of CAF activation, typically indicates a poor prognosis [37, 38]. Cav-1 tends to decrease during the oncogenic transformation of fibroblasts [39], and its loss of expression is usually associated with early recurrence, high metastatic risk, and drug resistance [40–44]. However, a few studies indicate that high Cav-1 expression contributes to cancer cell migration and invasion and is associated with low survival rates [45, 46]. The expression of PDGFR exhibits spatial heterogeneity: PDGFRβ drives angiogenesis and immune escape in the primary lesions and is associated with radiotherapy resistance and short survival [47–49], but becomes an independent factor for prolonged recurrence-free survival (RFS) in brain metastases [50]; high expression of PDGFRα in primary tumors increases the risk of central nervous system (CNS) recurrence [51], yet its absence predicts poor prognosis [52]. These contradictory results emphasize the multifaceted roles of CAFs in the development and prognosis of breast cancer, highlighting the individual heterogeneity of CAFs. Differences in the expression of CAF-related proteins among different molecular subtypes and stromal types of breast cancer further suggest their high heterogeneity [53–55].

The functional roles of CAFs in breast cancer are also dual: most CAFs exert pro-tumorigenic activity by promoting tumor growth, metastasis, angiogenesis, immune escape, treatment resistance, and ECM remodeling [56–59]. However, specific CAF subpopulations can also exert tumor-suppressive effects by activating anti-tumor immunity and secreting tumor-suppressive factors [60]. The contradictions in the clinical prognostic relevance and functional roles of CAFs indicate that relying solely on surface protein markers is insufficient to fully grasp the complex heterogeneity of CAFs. To reveal the precise characteristics of different CAF subpopulations, including gene expression profiles, functional features, and potential regulatory mechanisms, a deeper analysis of the heterogeneous cellular components of CAFs at the single-cell level is required.

### Heterogeneous CAF subpopulations in breast cancer

In recent years, considerable efforts have been made to understand the complex heterogeneity of CAFs in terms of function and other aspects. Studies have found that CAFs are not a homogeneous cell population, but are composed of highly heterogeneous subpopulations with distinct phenotypes, functions, and spatial distributions. An in-depth analysis of CAF heterogeneity is crucial for understanding their multidimensional roles in breast cancer progression (Table 1).

### CAF-S1 to CAF-S4

Early studies mainly relied on combinations of cell surface markers to distinguish different CAF subpopulations. The first study combining α-SMA, PDGFRβ, and S100A4/FSP1 confirmed that CAFs are not a homogeneous population but consist of functionally and phenotypically distinct subpopulations, providing early evidence for CAF heterogeneity [61]. Subsequently, based on the expression patterns of six CAF surface markers, Costa et al. distinguished four CAF subpopulations with different expression profiles in human breast cancer: CAF-S1 (FAP^High^SMA^Med−High^FSP1^Med^PDGFRβ^Med−High^CD29^Med^CAV1^Low^); CAF-S2 (FAP^Neg^SMA^Neg^FSP1^Med−High^PDGFRβ^Med^CD29^Low^CAV1^Low^); CAF-S3 (FAP^Neg^SMA^Neg^FSP1^Neg−Low^PDGFRβ^Neg^CD29^Med^CAV1^Neg^); CAF-S4 (FAP^Neg−Low^SMA^High^FSP1^Low−Med^PDGFRβ^Low−Med^CD29^High^CAV1^Low^) [8]. Among these, the CAF-S1 and CAF-S4 subpopulations, characterized by high α-SMA expression, are enriched in triple-negative breast cancer (TNBC) and HER2-positive breast cancer. CAF-S1 also expresses high levels of FAP and is often located near epithelial tumor cells, forming a dense cellular “barrier”, and promoting the formation of an immunosuppressive microenvironment and distant tumor recurrence via high expression of molecules such as Cadherin 11 and CD73 [8, 62, 63]. CAF-S2 and CAF-S3 are enriched in luminal breast cancer patients and healthy tissues, respectively [8]. Further studies have found that these four CAF subpopulations are also present in axillary metastatic lymph nodes of breast cancer, where CAF-S1 and CAF-S4 drive cancer metastasis through the CXCL12/TGFβ and NOTCH signaling pathways, respectively [64]. The team also demonstrated the existence of CAF-S1 to S4 subpopulations in high-grade serous ovarian cancer (HGSOC) [65]. It is worth noting that heterogeneity exists even within the well-defined CAF-S1 subpopulation.

Multiplex fluorescence immunohistochemistry, through combinations of FAP, α-SMA, and PDGFR, can identify more CAF subpopulations, and specific CAF subpopulations have significant prognostic significance [66], but still faces the problem of limited indicators in a single detection. The emergence of scRNA-seq has broken through the limitations of traditional low-throughput technologies. Focusing on the key pro-tumorigenic FAP^+^ CAF-S1 subpopulation, Kieffer et al. used scRNA-seq to conduct a deeper analysis. In 18,805 CAF-S1 cells from 8 primary breast cancers, 8 distinct subclusters were defined based on unique transcriptional signatures and functional pathway enrichment, belonging to the myofibroblastic CAFs (myCAFs) and inflammatory CAFs (iCAFs) categories. Among them, 5 myCAF clusters were characterized by high expression of genes coding ECM proteins (ECM-myCAF), TGFβ signaling pathway (TGFβ-myCAF), wound healing (Wound-myCAF), IFNαβ (IFNαβ-myCAF), and acto-myosin pathway (acto-myCAF), and 3 iCAF clusters were distinguished by detoxification pathway (Detox-iCAF), interleukin-signaling (IL-iCAF), and IFNγ (IFNγ-iCAF). The existence of the 5 most abundant CAF-S1 clusters (ECM-myCAF, TGFβ-myCAF, Wound-myCAF, Detox-iCAF, IL-iCAF) was confirmed in head and neck squamous cell carcinoma (HNSCC) and non-small cell lung cancer (NSCLC), revealing their pan-cancer conservation [67].

The application of spatial transcriptomics technologies further revealed that CAF subpopulations are not randomly distributed but exhibit highly structured spatial organization patterns closely linked to their functions [68]. Croizer et al. used spatially resolved technologies to identify 10 spatially organized FAP^+^ CAF-related cellular niches, termed EcoCellTypes (ECT) [5]. Specifically, Detox-iCAF and IL-iCAF are predominantly located in the peritumor conjunctive tissue; the ECM-myCAF are enriched in the tumor bed; TGFβ-myCAF are distributed both within the tumor and adjacent to normal lobules; Wound-myCAF reside in large nests of intra-tumoral stroma, while ECM-myCAF and IFNαβ-myCAF are commonly observed in tumor cell-enriched regions [5]. Spatial analysis also revealed interaction networks between CAF subpopulations and their neighboring cells. For example, ECM-myCAF, IFNαβ-myCAF, and TGFβ-myCAF clusters co-localize with immune cells such as TREM2^+^ tumor-associated macrophages (TAM) and SPP1^+^ TAM within the tumor bed, while Detox-iCAF co-localizes with FOLR2^+^ TAM in immuno-protective niches [5]. Additionally, single-cell spatial multi-omics revealed that s1-CAF neighborhoods create an immune-excluded microenvironment, leading to reduced T cell infiltration in the tumor bed. Conversely, s3-CAF neighborhoods, characterized by high infiltration of macrophages and neutrophils, exhibit elevated proportions of stress response T cells (Tstr), which correlate with T cell dysfunction and poor response to immune checkpoint therapies [3]. These studies suggest a close connection between spatial CAF subtypes and immune cell distribution, which synergistically regulate TIME.

Notably, as important complements to scRNA-seq and spatial transcriptomics technologies, high-dimensional spatial protein imaging technologies such as imaging mass cytometry (IMC) and multiplexed ion beam imaging (MIBI) have brought breakthroughs in in situ analysis of heterogeneous CAF subpopulations and their spatial landscapes within the complex TME at the protein level [69, 70]. These platforms can simultaneously image more than 40 protein markers on a single tissue section while preserving the spatial location information in the original tissue, which is crucial for resolving the high heterogeneity and spatial neighborhoods of CAFs [69, 70]. Røgenes et al. developed and validated a 42-marker IMC panel specifically for studying CAF niches in breast cancer, which included key CAF markers such as α-SMA, FAP, and PDGFR, as well as functional markers including YAP1 and pSMAD2 [71]. Their application of this panel to breast cancer tissues enabled the identification of as many as 19 distinct CAF subpopulations and revealed significant compositional differences of CAFs between luminal breast cancer and TNBC [71].

### MyCAFs, iCAFs, and apCAFs

Based on the difference in α-SMA expression levels, CAF-S1 can be further divided into myCAFs and iCAFs subpopulations: myCAFs exhibit high α-SMA expression and are spatially adjacent to tumor cells, while iCAFs have low α-SMA expression and are spatially distant from tumor cells, secreting large amounts of inflammatory factors such as IL-6 [72]. Additionally, iCAFs and myCAFs form distinct spatial modules, where iCAF subpopulations (such as IL-iCAFs, Detox-iCAFs, and IFNγ-iCAFs) tend to cluster together and enrich immune-related pathways, while myCAFs form a group that overlapped with elevated TGF-β signaling, dominating ECM remodeling and wound healing-related functions [73].

Sebastian et al. performed scRNA-seq in 4T1 tumors and identified six CAF subpopulations, including myCAFs, iCAFs, and CAFs expressing major histocompatibility complex (MHC) class II proteins [74]. Subsequently, Wu et al. also confirmed the existence of myCAFs and iCAFs in TNBC and identified two perivascular-like (PVL) subpopulations [75]. Single-cell transcriptomics, in vivo and in vitro studies revealed that CD26^+^ and CD26^−^ normal fibroblasts (NFs) were transformed into iCAFs and myCAFs, respectively [76]. Single-cell transcriptomics show that NFs clusters gradually disappear while CAFs clusters emerge during tumor development. Trajectory analysis indicates that both CD26^−^ and CD26^+^ NFs can first transition into iCAFs together and then further into myCAFs, with some CD26^−^ NFs directly transitioning into myCAFs. Clustree analysis further indicates CD26^+^ NFs tend to transition into iCAFs. These transitions occur during all stages of tumor development [76]. myCAFs often express high levels of ECM-related genes and are mostly distributed in invasive breast cancer (IBC), playing an important role in ECM production and remodeling; whereas iCAFs primarily express chemokines like CXCL12 and are present in higher proportions in DCIS, mainly involved in immunosuppression [5, 75, 77]. scRNA-seq also confirmed the existence of antigen-presenting CAFs (apCAFs) in breast cancer [78], which are similar to previously reported CAFs that can process and cross-present antigen, killing CD8⁺ T cells [79]. Notably, myCAFs, iCAFs, and apCAFs have previously been identified in pancreatic ductal adenocarcinoma (PDAC) [72, 80]. Furthermore, a recent pan-cancer study revealed four spatially distinct CAF subtypes, among which s1-CAFs and s2-CAFs showed consistent characteristics with myCAFs; s3-CAFs were enriched in iCAFs genes; and s4-CAFs showed high similarity to apCAFs [3], suggesting that these CAF subpopulations may have commonalities across different cancer types.

scRNA-seq also revealed CAF plasticity, with pseudotime analysis showing that CAF phenotypes can transition between iCAFs and myCAFs. This phenomenon was reported in both breast cancer and pancreatic cancer [5, 72, 81]. Wu et al. delineated five differentiation trajectories from iCAFs to myCAFs through pseudotime analysis and dissected their localization using integrated spatial transcriptomics, identifying “stromal-immune niches” and their roles in immune regulation [82].

Friedman et al. also confirmed that the ratio of S100A4^+^ and PDPN^+^ CAFs dynamically changes with tumor progression, with their functional programs transitioning from early immune-regulatory to wound-healing and antigen-presentation. This ratio change strongly correlates with BRCA mutational status and clinical outcomes [83]. PDGFRβ^+^ CAFs in tumors can be transformed from PDGFRα^+^ fibroblasts [84]. These dynamic changes suggest that CAF subpopulations may more accurately reflect different functional states of cells rather than fixed cell types.

### Vascular CAFs, matrix CAFs and developmental CAFs

Based on these observations, researchers have conducted more in-depth studies on CAF heterogeneity through scRNA-seq and spatial transcriptomics technologies to further reveal their functional characteristics and spatial distribution. Bartoschek et al. performed scRNA-seq on 768 transcriptomes of mesenchymal cells and defined three subpopulations of CAFs with different functions and origins: vascular CAFs (vCAFs), matrix CAFs (mCAFs), and developmental CAFs (dCAFs) [85]. Spatial proteomics also validated the existence of these subpopulations. Using IMC, Cords et al. designed a 41-plex antibody panel for breast cancer samples, which validated 7 CAF subpopulations including vCAFs and mCAFs at the protein level, providing direct spatial validation at the protein level for the functional phenotypes of CAFs defined by scRNA-seq [86]. Furthermore, a landmark IMC study involving over 1,000 NSCLC patients also confirmed the existence of subpopulations such as vCAFs and mCAFs, and clarified that their spatial distribution is strongly associated with immune infiltration and patient survival [87].

vCAFs within the stromal niche are tightly associated with blood vessels in early stages of tumors and gradually infiltrate into the stroma during tumor progression, which is closely related to angiogenesis. mCAFs specifically express transcripts of a large variety of ECM-related genes, including glycoproteins (Dcn, Lum, and Vcan), structural proteins (Col14a1), matricellular proteins (Fbln1, Fbln2, and Smoc), and matrix-modifying enzymes (Lox and Loxl1), and are found at high prevalence at the invasive front of tumors. In contrast, dCAFs are intermingled with the malignant epithelium during early stages of tumor development, and their gene profiles are associated with cell differentiation and tissue morphogenesis [85].

**Fig. 1:**
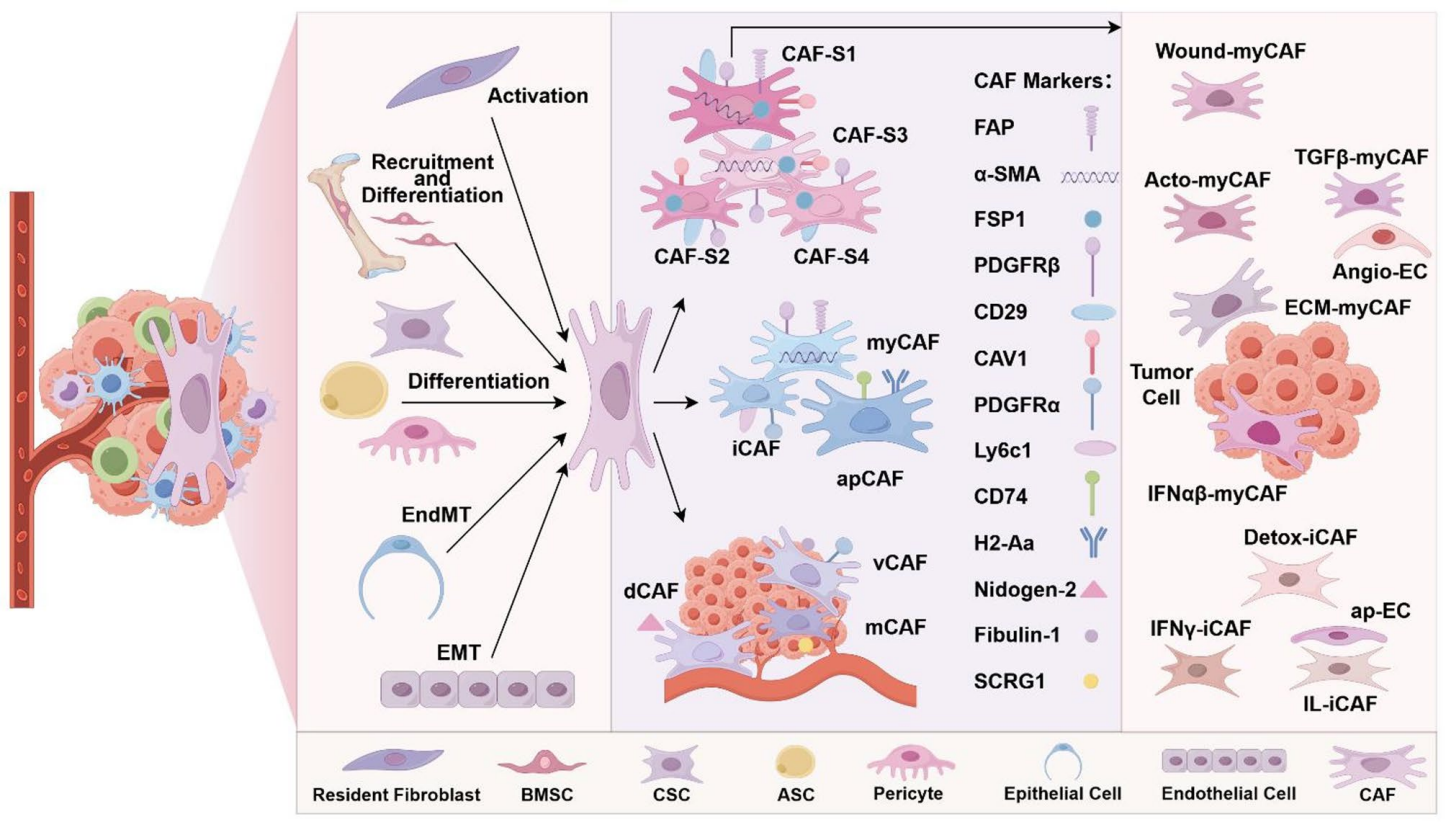
The origins, markers, and subpopulations of CAFs in breast cancer. The origins of CAFs include resident fibroblasts, bone marrow mesenchymal stem cells (BMSCs), cancer stem cells (CSCs), adipose-derived stem cells (ASCs), pericytes, epithelial cells, and endothelial cells. CAFs can be categorized into distinct subpopulations, such as CAF-S1 to CAF-S4, myCAF/iCAF/apCAF, and vCAF/mCAF/dCAF. CAF-S1 can be further subdivided into 8 distinct subclusters

In summary, scRNA-seq has revealed the diversity and dynamic changes of CAF subpopulations, while spatial transcriptomics technologies have further elucidated their spatial localization and functional characteristics, indicating the core roles of key CAF subpopulations in the TME (Fig. 1).

**Table 1:** CAF subpopulations in breast cancer

Subpopulations	Identification Methods	Markers (+)	Functions	Enriched Regions	Refs.
CAF-S1	FACS	FAP, SMA, FSP1, PDGFRβ, CD29, CAV1	Immunosuppression	TNBC	[8]
CAF-S2	FACS	FSP1, PDGFRβ, CD29, CAV1	Unknown	LuminalA BC	[8]
CAF-S3	FACS	FSP1, CD29	Unknown	Healthy Tissue	[8]
CAF-S4	FACS	FAP, SMA, FSP1, PDGFRβ, CD29, CAV1	Matrix Contraction, Oxidative Metabolism	TNBC, HER2^+^ BC	[8]
vCAFs	scRNA-seq	Nidogen-2, Notch3	Vascular Development and Angiogenesis	Proximity to the Vasculature	[85]
mCAFs	scRNA-seq	Fibulin-1, PDGFRα	ECM Synthesis and Remodeling, Immune Regulation	Invasive Front of Tumors	[85]
dCAFs	scRNA-seq	SCRG1	Cell Differentiation, Tissue Development and Morphogenesis	Tumor-Stroma Boundary	[85]
myCAFs	scRNA-seq, FACS, IHC	ACTA2, FAP, PDPN, COL1A1, TNC	ECM Synthesis and Remodeling, Tumor Invasion, Angiogenesis	Invasive Tumor Interface, Perivascular Stroma	[74–76]
iCAFs	scRNA-seq, FACS, FCM	PDGFRα, Ly6c1, IL-6, CXCL12	Immune Regulation	Distal Stroma, Immune-Infiltrated Regions	[74–76]
apCAFs	scRNA-seq, IF	CD74, H2-Aa	Antigen Presentation	Tumor-Stroma Boundary	[74]
ECM-myCAFs	scRNA-seq	ANTXR1, SDC1, LRRC15, COL1A2	ECM Remodeling, Immune Checkpoint Upregulation	Dense Stromal Areas with Collagen Deposition	[67]
TGFβ-myCAFs	scRNA-seq	ANTXR1, LAMP5, SDC1, TGFβ1, CST1	Activation of TGFβ Signaling Pathway, Immunosuppression	Treg-Enriched Immunosuppressive Niches	[67]
Wound-myCAFs	scRNA-seq	ANTXR1, CD9, SEMA3C, SFRP4	Wound Healing, Tumor Invasion, Stromal Remodeling	Wound-Like Stroma near Invasive Tumor Front	[67]
IFNαβ-myCAFs	scRNA-seq	IFIT3, IRF7	IFNαβ Signaling Pathway Involvement	IFN-Responsive Stromal Zones	[67]
Acto-myCAFs	scRNA-seq	GGH, PLP2	Actomyosin Complex Formation	High-Tension Stromal Regions	[67]
Detox-iCAFs	scRNA-seq	GPC3, DLK1	Detoxification, Inflammatory Response	Stromal Regions with High Detoxification Activity	[67]
IL-iCAFs	scRNA-seq	DLK1	Promotion of Pro-Inflammatory Microenvironment	Peritumoral Inflammatory Zones	[67]
IFNγ-iCAFs	scRNA-seq	CD74	Response to IFNγ Signaling, Antigen Presentation	Immune-Infiltrated Areas	[67]
pCAFs	scRNA-seq, FACS, IHC, IF	PDPN	Immune Regulation, Wound Healing	Cancer-Adjacent Regions	[83]
sCAFs	scRNA-seq, FACS, IHC, IF	S100A4	Antigen Presentation, Protein Folding, Metabolic Regulation	Dense Stroma	[83]
CD10^+^GPR77^+^ CAFs	FACS, IHC, IF	CD10, GPR77	Maintenance of CSC Stemness, Chemoresistance	Tumor Stroma	[180]

FACS: Fluorescence-activated cell sorting; IHC: Immunohistochemistry; FCM: Flow cytometry; IF: Immunofluorescence; BC: Breast cancer

## Crosstalk between CAFs and immune cells in breast cancer microenvironment

The heterogeneity of CAFs determines their complex immunomodulatory functions in the TME. CAFs significantly impact the infiltration and function of immune cells through multiple mechanisms, including secreting of cytokines and chemokines, and remodeling of ECM (Table 2). Different CAF subpopulations collaboratively construct an immunosuppressive network through unique molecular mechanisms, becoming a core driving force for tumor immune escape. A deeper understanding of the interaction mechanisms between CAFs, particularly their specific subpopulations, and immune cells within the breast cancer TME may offer new insights for potential immunotherapeutic strategies.

### Crosstalk between CAFs and T cells

CAFs exert inhibitory effects on T cells through various mechanisms in the breast cancer microenvironment. This section will discuss in detail the mechanisms by which CAFs affect T cells, focusing on four aspects: inhibiting T cell activation and proliferation, inducing T cell exhaustion and apoptosis, hindering T cell infiltration, and influencing T cell differentiation.

### Inhibition of T cell activation and proliferation

The arrangement and density of ECM can promote the activation of CAFs, which inhibit T cell activation and thereby accelerate breast cancer progression. In vitro 3D culture experiments demonstrated that when cultured in high-density and aligned ECM, CAFs can increase the expression of α-SMA and fibronectin while reduce the secretion of IL-2, thereby inhibiting T cell activation and accelerating breast cancer progression [88]. CAFs can also produce pro-inflammatory cytokines that promote the generation and expansion of Th17 cells, including IL-1, IL-6, IL-23, and TGF-β, which are key cytokines for human Th17 generation and differentiation [89]. TGF-β produced by CAFs can inhibit the cytotoxic activity of cytotoxic T lymphocytes (CTLs) by downregulating the expression of perforin, granzyme A/B, Fas ligand (FASL), and IFN-γ [90]. Histone deacetylase (HDAC) 6 is frequently upregulated in CAFs of breast tumors and promotes an immunosuppressive microenvironment. Genetic or pharmacological disruption of HDAC6 in CAFs inhibits the recruitment of regulatory T cells (Tregs) in tumors and increases the activation of CD8^+^ and CD4^+^ T cells in vivo [91]. In addition, both the immortalized human CAF lines from breast cancer and their parental CAFs are capable of effectively suppressing T cell proliferation [92]. Notably, this suppressive effect of CAFs on T cell proliferation has been shown to be more significant than the direct effect exerted by cancer cells themselves [93].

Various CAF subpopulations can also directly or indirectly inhibit T cell activation signals and proliferative capacity. In breast cancer, CAF-S1 recruits CD4⁺CD25⁺ T lymphocytes via CXCL12 secretion, followed by direct contact with T cells through surface molecules OX40L, PD-L2, and JAM2 to prolong their retention. CAF-S1 also utilizes B7H3, CD73, and DPP4 to enhance T cell survival while inhibiting their proliferative capacity [8]. CD73 is highly expressed in CAF-S1 and catalyzes the conversion of AMP into immunosuppressive adenosine to further suppress T cell activity [63]. A similar multi-step mechanism is also observed in ovarian cancer, where CAF-S1 attracts CD4^+^CD25^+^ T lymphocytes via CXCL12 secretion and enhance Treg-mediated inhibition of T effector proliferation through B7H3, CD73, and IL-6 [65]. The Ly6C⁺ pCAFs (PDPN^+^ CAFs) subpopulation significantly inhibits the proliferation and activation of CD8^+^ T cells by downregulating the expression of surface activation markers CD25 and CD69 [83]. Another population of FAP⁺PDPN⁺ CAFs, which closely interacts with T cells at the tumor periphery, was shown to significantly suppress the proliferation of CD4⁺ and CD8⁺ T cells by secreting nitric oxide [94]. Furthermore, ECM-myCAF isolated from HGSOC reduces CD8^+^ T cell cytotoxicity via the YAP1/TEAD signaling pathway [95]. Pan-cancer analysis indicates that both myCAFs and iCAFs can suppress T cell activity by secreting TGF-β [78]. C5 and TGF-β2 secreted by iCAFs have also been shown to contribute to the inhibition of T cell activity [75]. Notably, although apCAFs express MHC class II molecules and have the potential for antigen presentation [74], the lack of classical co-stimulatory molecules such as CD80 and CD86 may contribute to immune suppression by inducing either anergy or differentiation into Tregs [80]. Taken together, these mechanisms demonstrate that CAFs significantly impair the anti-tumor function of T cells through both chemical signals and physical contact.

### Induction of T cell exhaustion and apoptosis

T cell exhaustion is one of the key mechanisms of tumor immune escape, characterized by a sustained downregulation of T cell function and high expression of immune checkpoint molecules such as PD-1 and CTLA-4, with CAFs playing an important role in this process. It has been reported that CAFs kill CD8^+^ T cells in an antigen-specific and antigen-dependent manner via PD-L2 and FASL, and neutralizing PD-L2 or FASL can reactivate the cytotoxic capacity of T cells in vitro and in vivo [79]. In addition, IL-6 levels in CAF-conditioned media (CM) are significantly higher than that in NFs. CM with high IL-6 levels induces PD-L1 expression through the STAT3 and AKT pathways, and breast cancer cells treated with high IL-6 CM exhibit resistance to chimeric antigen receptor T-cell (CAR-T) cell killing [96]. When co-cultured with CD4^+^CD25^+^ T cells, CAF-S1 significantly enhances the percentages of Tregs expressing PD-1 and CTLA-4 [63]. The myCAF subpopulation is also deeply involved in inducing T cell exhaustion. TGFβ-myCAF often co-localizes with FOXP3^+^ Tregs in the TME and significantly upregulates the expression levels of PD-1 and CTLA-4 of these Tregs [5]. Similarly, ECM-myCAF not only promotes PD-1/CTLA-4 expression on Tregs, but also found to increase the percentage of FOXP3^+^ T cells and upregulate their checkpoint molecule levels. In turn, Tregs induce TGFβ-myCAF differentiation, forming a positive feedback loop of immunosuppression [67]. Additionally, study indicates that myCAFs potentially induce T cell exhaustion via the PD-1/PD-L1 axis [75]. iCAFs can also promote the expression of immune checkpoint molecules such as PD-1 on T cells [78], and the gene signature of iCAFs is significantly correlated with CTL dysfunction [75]. Moreover, apCAFs interact with T cells through the LGALS9-TIM-3/SORL1 pathway, inducing T cell apoptosis or functional inactivation [78]. These findings indicate that CAFs collectively promote T cell exhaustion through multiple mechanisms, providing support for tumor immune escape.

### Impeding T cell infiltration

Effective anti-tumor immunity requires T cell infiltration into the tumor parenchyma, and the presence of CAFs is closely associated with significant changes in T cell numbers. Studies show that CAFs lead to a decrease in the number of CD8^+^ T cells with critical anti-tumor function in the TME [97–99], reduced PD-L1 expression [98], and render tumors resistant to immune checkpoint blockade (ICB) therapy [99]. The reduction in CD8^+^ T cell numbers is associated with the upregulation of biglycan [100] and chitinase 3-like protein 1 (Chi3L1) [101] expression in CAFs, and is closely related to key biological processes such as EMT and immune regulation [97]. Concurrently, CAFs also recruit CD4^+^CD25^+^ T cells to the TME by secreting CCL5, and these T cells stimulate metastatic progression of breast cancer through the RANKL-RANK signaling pathway [102].

One of the core mechanisms by which CAFs impede T cell infiltration is the construction of physical barrier, with myCAFs being the primary contributors. The ECM-myCAF subpopulation highly expresses collagen and α-SMA. By secreting and remodeling the ECM extensively, they form a dense fibrotic matrix structure that physically hinders the infiltration of CD8^+^ T cells and other immune cells into the tumor core [5, 67]. Similarly, the TGF-β signaling-driven LRRC15^+^ CAF subpopulation in PDAC also forms a physical barrier by enhancing ECM deposition, restricting T cell infiltration into the tumor parenchyma, leading to an “immune exclusion” phenotype, and is directly associated with anti-PD-L1 therapy resistance [103]. Moreover, iCAFs specifically express hyaluronan synthases (HAS1, HAS2) to produce abundant hyaluronan (HA), forming a viscous stromal environment that also constitutes a physical barrier restricting T cell migration [80]. The presence of differentiated-PVL (dPVL) subpopulation is also significantly associated with lower CTL infiltration levels and density [75].

Beyond physical barriers, CAFs can regulate the spatial distribution of T cells through chemical signals. FAP^+^ CAFs, as the primary source of CXCL12 within tumors, secrete CXCL12 that enriches on the tumor cell surface. This forms a chemokine gradient via the CXCR4 receptor, repelling T cells to the tumor periphery and thereby reducing T cell infiltration [104, 105]. Inhibition of CXCR4 (such as using AMD3100) can reverse this effect, promoting T cell infiltration and enhancing the efficacy of anti-PD-L1 therapy [104, 105].

However, not all CAFs inhibit T cell infiltration. Only specific FAP⁺ CAF clusters, characterized by ECM accumulation, wound healing, and TGF-β signaling, are associated with T cell infiltration [106]. For instance, the IFNγ-iCAFs in breast cancer highly expresses EMILIN1, which locally modulates the immunosuppressive function of TGF-β and promotes CD8^+^ T cell infiltration [73]. Moreover, the Detox-iCAF subpopulation identified in HGSOC significantly enhances CD8^+^ T cell migration, functionally antagonizing ECM-myCAF [95]. More intriguingly, upon TGF-β signaling inhibition in breast cancer model, although the numbers of myCAFs and vCAFs decrease, a new subpopulation of CAFs, interferon-licensed CAFs (ilCAFs), appears [107]. These ilCAFs highly express CXCR3 ligands, which can effectively attract effector T cells infiltration into the tumor core via the CXCR3 receptor, and secrete immune-activating molecules to remodel the TME into an immune-permissive state [107]. Additionally, pan-cancer analysis shows that s1-CAF enrichment creates an immune-excluded microenvironment, leading to reduced T cell infiltration in the tumor bed and increased proportions of CD4^+^ Tregs and CD8^+^ exhausted T cells (Tex). The s3-CAF neighborhood exhibits a higher proportion of Tstr, while the s4-CAF neighborhood is enriched in CD4^+^ naive T cells (Tn), follicular helper T cells (Tfh), and central memory T cells (Tcm) [3].

### Influencing T cell differentiation

As central regulators of immune polarization in the breast cancer TME, CAFs shape an immunosuppressive phenotype through multiple mechanisms: beyond promoting the generation and function of Tregs, they modulate T-cell differentiation, thereby skewing the balance between effector and regulatory T-cells [93]. Studies show that elimination of CAFs in the 4T1 murine model shifts the immune microenvironment from Th2 to Th1 polarization, suppresses Treg recruitment, and enhances CD8^+^ T cell infiltration [108]. This regulatory function is closely related to the ability of CAFs to promote type 2 immunity and Treg subtype [92]. CAFs can also induce the differentiation of CD73^+^ γδ Tregs via the IL6/STAT3 pathway, and these Tregs mediate stronger immunosuppressive activity through adenosine production [109].

Different CAF subpopulations synergistically promote the formation of an immunosuppressive microenvironment through distinct mechanisms, with CAF-S1 being particularly prominent in this regard, significantly increasing the proportion of CD4⁺CD25⁺FOXP3⁺ Tregs through a multi-step mechanism and enhancing their ability to inhibit effector T cell proliferation [8, 65]. TGFβ-myCAF co-localize with FOXP3^+^ Tregs in the TME and recruit Tregs, which correlates with the enrichment of Tregs in tumors [5, 67]. ECM-myCAFs can also increase the percentage of FOXP3^+^ Tregs [67]. iCAFs secrete inflammatory factors such as IL-6, CXCL12, and CCL2 to recruit Tregs and B cells, promoting the formation of an immunosuppressive microenvironment [74, 75, 80]. Although apCAFs possess antigen-presenting potential, their lack of co-stimulatory molecules leads to a tendency to induce Treg differentiation when interacting with T cells [80]. In addition, apCAFs co-localize with Tregs spatially and may enhance Treg immunosuppressive function [78]. These mechanisms collectively reveal how CAFs systemically reshape the TME into an immunosuppressive phenotype by regulating T cell differentiation lineages, thereby providing a protective microenvironment for tumor progression.

### Crosstalk between CAFs and macrophages

In breast cancer TME, the interaction between CAFs and TAMs also promotes tumor progression through multiple mechanisms. CAFs finely regulate the recruitment, polarization status, and functional activity of macrophages by secreting various cytokines and chemokines [110]. In-depth analysis of the interaction mechanisms between CAFs and TAMs is also crucial for developing innovative treatment strategies for breast cancer.

### CAFs promote TAM recruitment

CAFs play a critical role in shaping the immune landscape of the TME, with one of their key functions being the efficient recruitment of monocytes to the tumor area and the establishment of pro-tumoral M2-like TAMs [111]. High CAF activation levels positively correlate with increased TAM infiltration in TNBC patients [112]. CAFs mainly recruit monocytes and macrophages by secreting specific chemokines. Among these, CXCL12 (SDF-1) is a key molecule, and CXCL16 can also chemotactically recruit monocytes to breast tumor tissues [113–115]. High expression of CXCR4 is also associated with increased TAM infiltration in tumor tissues [116]. It is worth noting that specific CAF subpopulations play a prominent role in this process. For example, iCAFs are shown to recruit monocytes via the CXCL12-CXCR4 axis and reprogram them into STAB1⁺TREM2^high^ lipid-associated macrophages (LAMs) with potent immunosuppressive functions [117]. Similarly, iCAFs derived from CD26^+^ NFs also recruit CD11b^+^ monocytes in a CXCL12-dependent manner [76]. Furthermore, soluble factors secreted by breast cancer cells can stimulate NFs to express CXCR4 and transdifferentiate into CAFs, promoting the transport of macrophages to tumor sites [118]. CCL2 is another important chemokine secreted by CAFs. It recruits monocytes, particularly inflammatory monocytes, by binding to its receptor CCR2, promoting their infiltration into primary and metastatic tumor sites and differentiation into M2 macrophages [119–121]. The absence of TGF-β type 2 receptor in fibroblasts leads to increased CCL2 secretion and enhances tumor progression associated with TAM recruitment [122]. In addition to CXCL12 and CCL2, CAFs also utilize other signaling molecules to regulate TAM recruitment. IL-6 secreted by CAFs not only induces the differentiation of macrophages [89], but its inhibition also reduces CCL2 secretion and subsequent monocyte recruitment [119]. CAF-derived IL-33 also significantly enhances the recruitment of TAMs [123]. iCAFs can also secrete molecules like C5 and TGF-β2 to activate myeloid cells including TAMs [75]. Certain CAF subpopulations, such as Detox-iCAF, IL-iCAF, and IFNγ-iCAF, recruit monocytes by secreting chemokines and induce their polarization toward a FOLR2^+^ TAM phenotype [5]. CAFs can also indirectly promote myeloid cell recruitment through ECM modification, for instance, macrophages tend to traffic to HA-rich stromal regions [124].

### CAFs induce TAM polarization

CAFs not only induce the recruitment of TAMs to tumor tissues, but more crucially, they drive the polarization of these cells toward the M2-like phenotype through multiple mechanisms. Even existing M1 macrophages exhibit increased expression of M2 markers under the action of CAFs [115]. Several factors secreted by CAFs are involved in the regulation of M2 polarization, such as IL-4, IL-6, and TGF-β, thereby promoting tumor growth and metastasis [108, 125, 126]. CAFs are also capable of producing high levels of IL-33, which not only significantly enhances the transition of TAMs to the M2-like phenotype, but also activates the NF-κB-MMP9-laminin pathway through the ST2 receptor to regulate tumor stroma-mediated metastasis [123]. TGF-β is another potent immunoregulatory factor released by CAFs, capable of suppressing both innate and adaptive immune responses against breast cancer cells [127, 128]. Macrophages exposed to TGF-β1 acquire an M2-like phenotype and exhibit various tumor-promoting functions [128]. The myCAF subpopulation utilizes the TGF-β1 signaling pathway to promote an immunosuppressive phenotype of myeloid cells, including TAMs, thereby supporting tumor progression [75]. In addition to secreted factors, HDAC6 in CAFs regulates the immunosuppressive properties of TAMs by modulating the STAT3-COX2 pathway. Inhibition of HDAC6 reduces M2-associated gene expression in macrophages and drives their polarization toward the M1 phenotype [91]. Chi3L1, which is highly upregulated in CAFs of murine breast tumors, also induces M2 polarization of TAMs. Genetic ablation of Chi3L1 suppresses tumor growth, macrophage recruitment, and reprogramming to the M2-like phenotype [101]. Moreover, the interaction between CAFs and TAMs is not unidirectional but involves a complex positive feedback loop. For example, macrophages exposed to TGF-β acquire an M2-like phenotype, while M2-like TAMs secrete TGF-β to not only promote EMT in tumor cells but also promote CAF activation [128, 129]. Additionally, apCAFs promote macrophage polarization to a pro-inflammatory phenotype, and these polarized macrophages in turn boost apCAF generation, establishing a positive feedback loop that intensifies anti-tumor immunity [130].

### Crosstalk between CAFs and MDSCs

The concept of MDSCs was proposed in 2007 [131], and they possess potent immunosuppressive activity in the breast cancer TME [132]. Gunaydin et al. identified a novel MDSC subset that shares phenotypic and functional similarities with CAFs in breast cancer tissues, suggesting a potential correlation between MDSCs and CAFs [133]. CAFs are key mediators of MDSC recruitment in TME, and the chemokine CXCL1 secreted by them plays an important role in recruiting MDSCs to tumor tissues [134]. In the TNBC microenvironment, CAFs highly express the chemokine CXCL16, which attracts monocytes and drives their differentiation into MDSCs [114]. Co-culture experiments of CAFs and MDSCs also show that when CAFs are exposed to estrogen, they secrete SDF-1α, directly promoting the recruitment of MDSCs to the TME [135]. Another study has confirmed that CAFs directly recruit MDSCs by secreting CCL2, promoting their accumulation in the TME, and promoting MDSC differentiation and immunosuppressive function through cytokines such as GM-CSF, IL-4, and IL-13 [136]. It has also been reported in various solid tumors that CCL2 secreted by CAFs recruits MDSCs via the STAT3-CCL2 signaling pathway, thereby promoting tumor growth [137, 138].

### Crosstalk between CAFs and NK cells

Natural killer (NK) cells, defined by Herberman et al. in 1976 [139], primarily exert innate cytotoxic functions in the TME [140]. Analysis of samples from breast cancer patients shows that NK cells are enriched in stroma-rich regions, and the upregulation of NK-binding ligands on CAFs inhibits NK cell cytotoxicity against cancer cells through ligand-receptor interactions [141]. In addition, CAFs can secrete large amounts of TGF-β, blocking the activation and cytotoxicity of NK cells through multiple pathways [142]. TGF-β in tumor models drives the transdifferentiation of NK cells into type 1 innate lymphoid cells (ILC1s) lacking cytotoxic function, thereby facilitating immune escape [143]. It has also been reported that TGF-β inhibits NK cell-mediated IFN-γ production and antibody-dependent cellular cytotoxicity (ADCC) by inducing SMAD3 phosphorylation [144]. Du et al. identified a novel PDPN^+^ CAF subpopulation in HER2-positive breast cancer, which inhibits NK cell-mediated ADCC by secreting immunosuppressive factors IDO1 and TDO2 [145]. ECM-myCAF and TGFβ-myCAF subpopulations also collectively inhibit NK cell cytotoxicity by downregulating perforin and granzyme B expression and upregulating the proportion of NKG2A^+^ NK cells, further contributing to the establishment of an immunosuppressive microenvironment [5].

### Crosstalk between CAFs and other immune cells

In addition to the above-mentioned cells, the crosstalk between CAFs and other immune cells also plays an important role in regulating the TIME of breast cancer. Mast cells (MCs), as important effector cells of innate immunity, their interaction with CAFs has gradually attracted attention. IL-6 is considered a chemoattractant for MCs, capable of stimulating MC migration [146], and promoting MC growth through a fibroblast-dependent mechanism [147]. Hugo et al. demonstrated that MCs promote CAF proliferation by secreting IL-6, and CAFs in turn secrete IL-6 to further stimulate MC activation and proliferation, forming a bidirectional positive feedback loop that collectively promotes tumor immune escape and malignant progression [148].

Dendritic cells (DCs), as specialized antigen-presenting cells connecting innate immunity and adaptive immunity [149], have their biology potentially affected by the CAF secretome in several ways [150]. By secreting TGF-β, IL-6, and VEGF, CAFs inhibit DC maturation and antigen-presenting function through multiple mechanisms [7]. Moreover, fibroblasts can significantly increase the total number of DCs and the expression levels of both co-inhibitory and co-stimulatory molecules on DCs, endowing DCs with tolerogenic features [151].

In addition, a retrospective study showed that the presence of CAFs in the breast cancer TME is significantly associated with low tumor-infiltrating lymphocytes (TILs) and reduced memory B-cell levels [98]. CAFs can also promote B cell survival and maturation via the TNFSF13B-TNFRSF13B pathway, enhancing the immunosuppressive microenvironment, and recruit plasmacytoid DCs (pDCs) via the CXCL12-CXCR3 axis. These DCs secrete granzyme B to inhibit T cell proliferation [78]. The s4-CAF subpopulation also interacts frequently with B cells and support the differentiation and function of B cells through JAG1-NOTCH2 and LGALS9-CD47 interactions. Besides, the interaction between s4-CAFs and plasma cells facilitates plasma cell adhesion and signal transduction, and promotes the maintenance of the plasma cell niche [3].

Furthermore, CAFs have been reported in hepatocellular carcinoma to recruit neutrophils by secreting SDF-1α [152] and induce the polarization of N2 phenotype neutrophils by upregulating CXCL6 and TGF-β expression in tumor cells, thereby promoting tumor progression [153]. Pan-cancer analysis reveals that s3-CAFs interact with neutrophils through CXCL12-CXCR4 and ANXA1-FPR1, facilitating neutrophil infiltration and modulating their activation and function [3]. However, the above-mentioned mechanisms have not yet been confirmed in the breast cancer microenvironment, suggesting that their potential regulatory roles need further investigation.

**Fig. 2:**
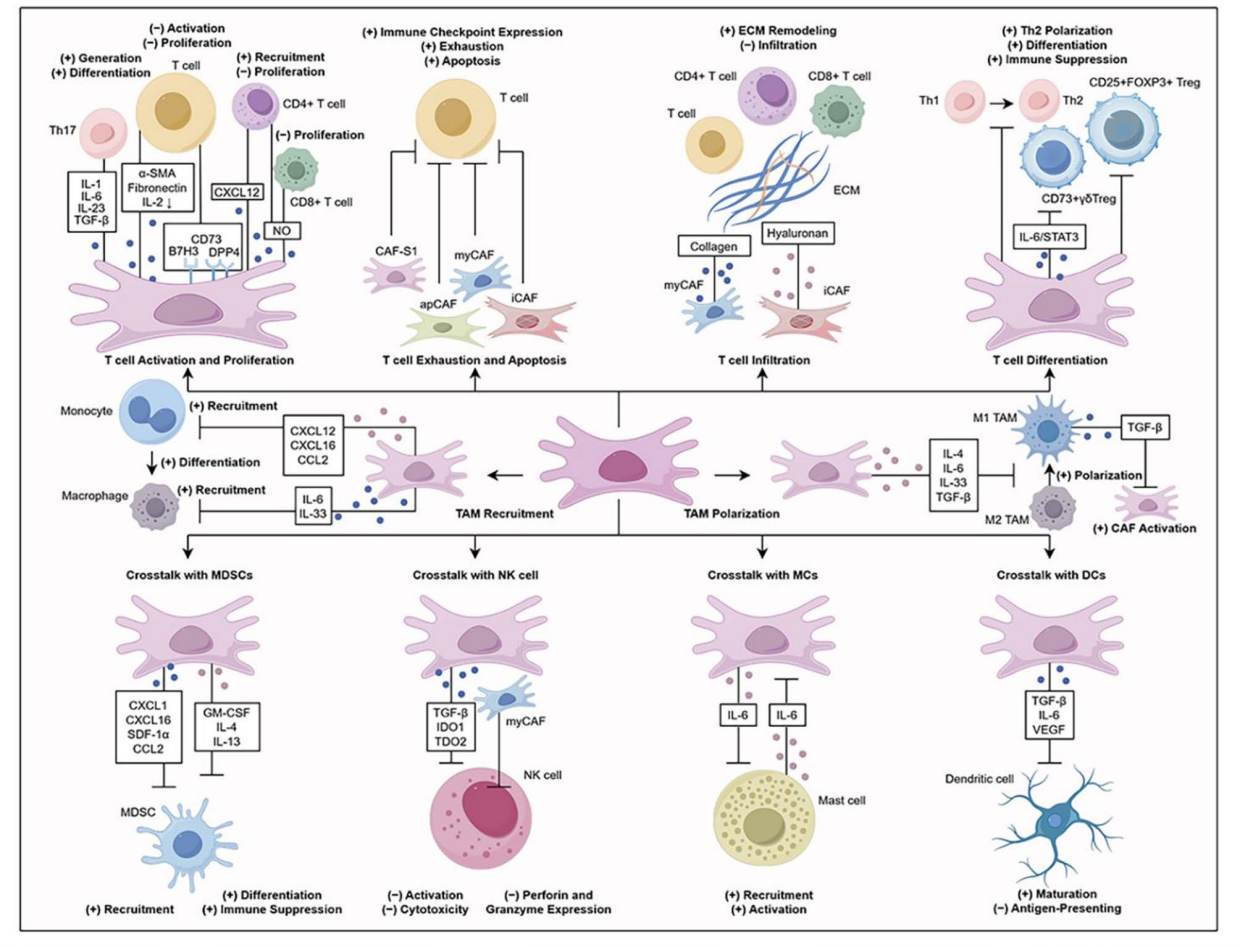
Crosstalk between CAFs and immune cells in breast cancer microenvironment. CAFs shape an immunosuppressive microenvironment through crosstalk with immune cells. Specifically, CAFs inhibit T cell activation and proliferation, induce T cell exhaustion and apoptosis, impede T cell infiltration, affect T cell differentiation, promote the recruitment and polarization of TAMs toward an M2-like phenotype, and engage in crosstalk with MDSCs, NK cells, MCs, and DCs

In summary, CAFs are not a homogeneous population, and complex communication networks exist between their different subpopulations and immune cells, playing multifunctional roles in shaping the TME, thereby shaping the characteristics of immune cells and the broader tumor immune landscape (Fig. 2). The plasticity and functional heterogeneity of CAFs provide opportunities for therapeutic intervention. In-depth analysis of these mechanisms will help uncover the molecular basis of tumor immune escape and provide a robust theoretical foundation for developing CAF-targeted immunotherapeutic strategies.

**Table 2:** Overview of effects of CAFs on immune cells in breast cancer

Secretory Cells	Stimulating Factors	Target Immune Cells	Mechanisms	Biology Effects	Refs.
CAFs	α-SMA, Fibronectin	T cells	Trigger integrin-mediated mechanical transduction	Inhibit T cell activation	[88]
FAP⁺PDPN⁺ CAFs	Nitric Oxide	CD4^+^ T cells, CD8^+^ T cells	Alter T cell function through protein modifications	Suppress CD4⁺ and CD8⁺ T cell proliferation	[94]
CAF-S1	CD73, Adenosine	CD4⁺CD25⁺FOXP3⁺ Tregs	Promote PD-1/CTLA-4 expression on Tregs	Promote Treg activation and suppressive function	[63]
apCAFs	LGALS9	CD4^+^ T cells, CD8^+^ T cells	LGALS9-TIM-3/SORL1 pathway	Induce T cell exhaustion	[78]
myCAFs	Collagen, α-SMA	T cells	Secretion and remodeling of the ECM	Impede T cell infiltration	[5, 67]
CAFs	IL-6	CD73⁺ γδ Tregs	IL-6/STAT3 pathway	Induce CD73⁺ γδ Treg differentiation	[109]
CAF-S1	CXCL12	CD4⁺CD25⁺ T cells	Cell contact retention via OX40L/PD-L2/JAM2, differentiation induction via B7H3/CD73/DPP4	Promote CD25⁺FOXP3⁺ Treg differentiation	[8]
CAFs	CXCL12, CCL2	Monocytes	CXCL12-CXCR4 axis, CCL2-CCR2 axis	Promote TAM recruitment	[115, 121]
CAFs	TGF-β	Macrophages	Induce SNAIL expression via SMAD2/3 and PI3K/AKT pathways	Induce TAM polarization into the M2 phenotype	[126]
CAFs	CXCL1	MDSCs	CXCL1-CXCR2 axis	Increase MDSC infiltration	[134]
CAFs	TGF-β	NK cells	Induce SMAD3 phosphorylation, inhibit IFN-γ expression	Inhibit NK cell cytotoxicity	[144]
PDPN^+^ CAFs	IDO1, TDO2	NK cells	Tryptophan depletion	Inhibit NK cell-mediated ADCC	[145]
CAFs	IL-6, SCF	MCs	Induce STAT3 phosphorylation	Stimulate MC activation and proliferation	[148]
CAFs	TGF-β, IL-6, VEGF	DCs	Downregulation of MHC class II molecules on DCs	Inhibit DC maturation and antigen-presenting function	[7]
CAFs	TNFSF13B	B cells	TNFSF13B-TNFRSF13B pathway	Promote B cell survival and maturation	[78]
CAFs	CXCL12	pDCs	CXCL12-CXCR3 axis	Recruit pDCs and inhibit T cell proliferation	[78]

## Therapeutic strategies targeting CAFs in breast cancer

**Fig. 3:**
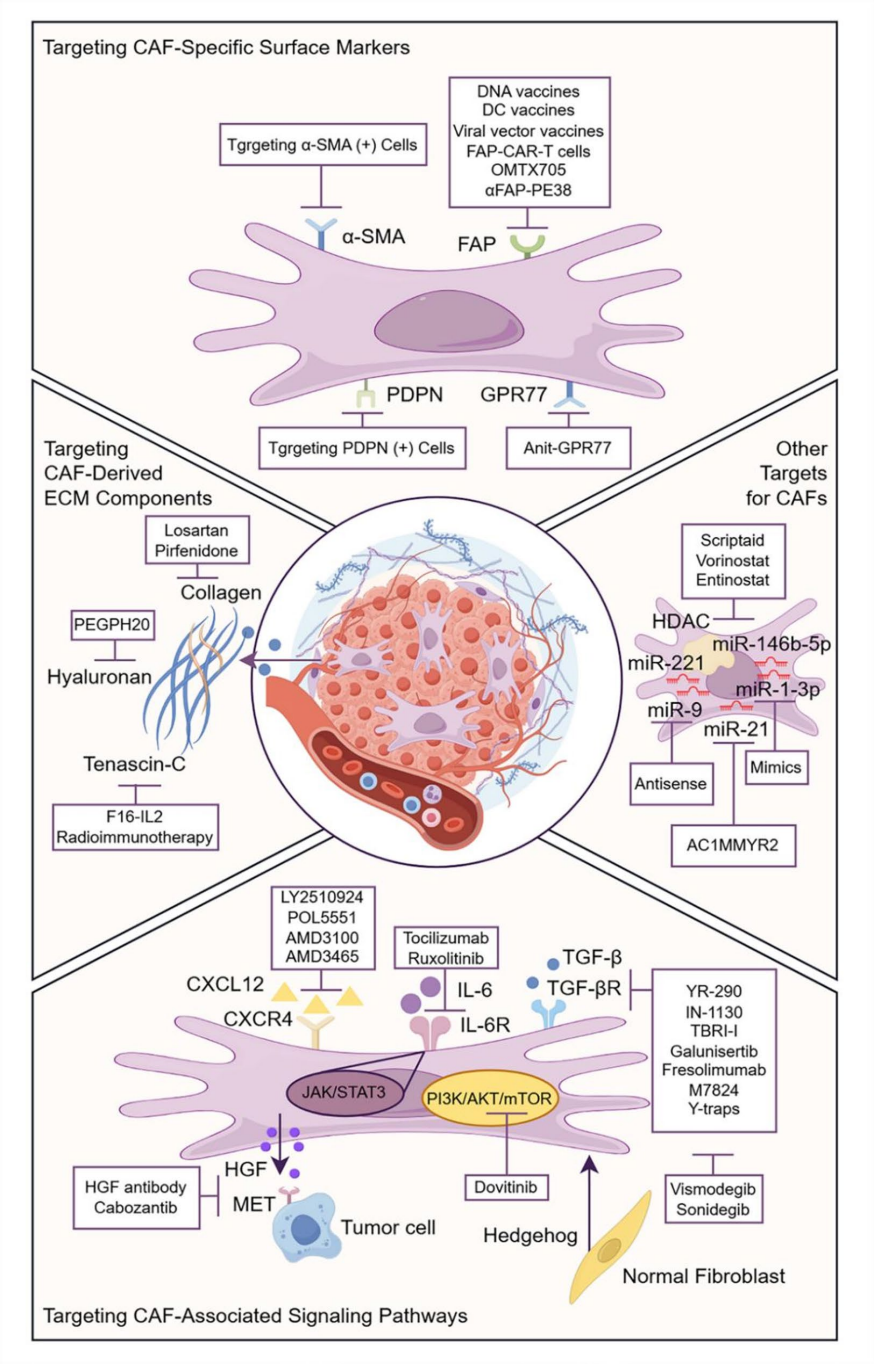
Therapeutic strategies targeting CAFs in breast cancer. CAFs can be directly depleted by targeting specific surface markers such as FAP and α-SMA; the activation or function of CAFs can be inhibited by targeting CAF-associated signaling pathways; ECM components derived from CAFs, such as collagen and HA, can be targeted to enhance immune cell infiltration; furthermore, epigenetic modulators targeting HDAC and multiple miRNAs also provide directions for CAF-targeted breast cancer therapy

Given that stromal cells exhibit higher genetic stability compared to cancer cells [154], the focus of cancer therapy is gradually shifting from “cancer cell-centric” to “stroma-centric” (Fig. 3). In this context, targeting CAFs has emerged as a highly promising therapeutic strategy in the field of breast cancer, aiming to fundamentally eliminate the complex pro-tumorigenic effects of CAFs and possessing significant potential for clinical translation.

### Targeting CAF-specific surface markers

The core of this strategy is to utilize molecular markers specifically expressed on the surface of CAFs to guide the immune system or therapeutic drugs to precisely identify and act on CAFs in the TME, thereby achieving direct killing or elimination of them. Currently, various CAF surface markers have been explored as therapeutic targets.

FAP is one of the most widely studied targets. As a specific marker of CAFs, it is highly expressed in CAFs of over 90% of epithelial-derived tumors, including breast cancer, while its expression is limited in normal tissues [155, 156], making it an ideal therapeutic target. Studies have shown that the elimination of FAP^+^ cells can effectively alleviate the immunosuppressive state within the TME, enhance the function of CD8^+^ T cells, and thereby prolong the survival of tumor-bearing mice [136]. Therapies targeting FAP are diverse, among which FAP vaccines are designed to induce a specific immune response targeting FAP to eliminate CAFs, with specific forms including DNA vaccines, DC vaccines, and viral vector vaccines. DNA vaccines expressing FAPα successfully reduced tumor growth in the 4T1 breast cancer model by stimulating FAPα-specific CTL responses [157]. Optimized FAP DNA vaccines further improved the anti-tumor effect by enhancing antigen secretion and immunogenicity [158]. DC vaccines are also considered an effective strategy [159]. DC vaccines transfected with FAP mRNA exhibited tumor-inhibiting effects in multiple tumor models [160], while composite DC vaccines can induce broad T cell responses and greater antitumor activity [161]. Furthermore, the exosome-like nanovesicles (eNVs)-FAP vaccines derived from FAP gene-engineered tumor cells have also demonstrated promising antitumor potential in preclinical studies [162]. Combination therapy strategies, such as FAP vaccines combined with cyclophosphamide [163] or tumor antigen vaccines [164], can further enhance therapeutic effect. FAP-targeted therapy based on CAR-T demonstrated significant efficacy by directly killing FAP^+^ CAFs and enhancing the antitumor response of CD8^+^ T cells [105, 165]. Photo-immunotherapy holds promise for further improving the efficacy and safety of FAP-CAR-T cell therapy [166]. Antibody-drug conjugates (ADCs), such as OMTX705, can selectively deliver drugs to FAP^+^ CAFs, showing complete tumor growth inhibition when combined with chemotherapy [167]. Immunotoxins, as another option for targeting FAP, can selectively deliver toxins to FAP^+^ CAFs and induce their apoptosis. For example, αFAP-PE38 inhibited the infiltration of immune cells by depleting FAP^+^ stromal cells in a breast cancer model, and effectively suppressed tumor growth in vivo when combined with paclitaxel [168]. Other emerging FAP-targeting strategies, such as immunocytokines [169, 170], nanotherapies [171, 172], bispecific liposomes [173], and oncolytic adenovirus [174], have all shown certain therapeutic potential. However, it is important to note that FAP is also expressed in stromal cells of normal tissues, and thus FAP-targeted therapy may induce side effects such as cachexia, anemia, and bone toxicity [175, 176]. This highlights that therapies targeting the tumor stroma must possess high specificity to spare healthy tissues. Therefore, in-depth analysis of heterogeneous subpopulations of CAFs is crucial for developing more precise and safer targeted treatment strategies.

In addition to FAP, other CAF surface markers are also being actively explored as therapeutic targets. For instance, specific nanoparticles targeting α-SMA^+^ cells can improve drug delivery efficiency within the tumor or enhance immune responses, thereby inhibiting tumor progression [177, 178]. Therapeutic strategies targeting PDPN have shown effects of enhancing anti-tumor immunity and prolonging survival of mice in preclinical models [179]. Additionally, targeting specific CAF subpopulations associated with treatment resistance, such as CD10^+^GPR77^+^ CAFs, can not only effectively inhibit tumor growth, but also restore tumor sensitivity to chemotherapy, representing a highly promising new therapeutic direction [180].

### Targeting CAF-associated signaling pathways

CAFs profoundly influence the initiation, progression, invasion, and metastasis of breast cancer by secreting various molecules and activating specific signaling pathways. Therefore, precisely analyzing and targeting key signaling pathways related to CAFs has become an important research direction for developing novel breast cancer therapies.

First, CAFs can promote breast cancer invasion through the TGF-β signaling pathway [181], and intervention in this pathway is a promising therapeutic approach [182]. Current strategies targeting the TGF-β signaling pathway mainly include small molecule inhibitors and monoclonal antibodies. Small molecule inhibitors often target the TGF-β type I receptor (TGF-βRI), such as IN-1130 and YR-290, which can significantly inhibit the invasion and metastasis of breast cancer and prolong the survival of tumor-bearing mice [183, 184]. Systemically administered TGF-βRI inhibitors, such as TBRI-I, also showed potential to inhibit lung and bone metastases in animal models [185]. Additionally, the TGF-β pathway inhibitor galunisertib can reverse the suppressive state of T cells, and its combination with PD-L1 blockade mediates a stronger anti-tumor immune response [186]. The monoclonal antibody fresolimumab can neutralize all three isoforms of TGF-β, and a survival benefit was observed in high-dose group when combined with radiotherapy in a clinical trial for metastatic breast cancer [187, 188]. Inhibition of the TGF-β pathway may also enhance drug penetration into tumor tissues by improving the physical barrier of the TME [189–191]. However, the limited clinical success of anti-TGF-β drugs has driven the development of bifunctional drugs, such as bintrafusp alfa (M7824) [192] and bifunctional antibody-ligand traps (Y-traps) [193], which simultaneously block PD-L1 and TGF-β signals. The development of these drugs embodies the concept of “multi-pathway synergistic intervention” and is expected to further improve the efficacy of immunotherapy.

The CXCL12-CXCR4 signaling axis is another key pathway through which CAFs mediate tumor progression [194], and blocking the interaction between CXCL12 and its receptor CXCR4 can exert anti-tumor effects. A variety of CXCR4 antagonists, such as LY2510924 [195], POL5551 [196], AMD3100 [197], and AMD3465 [198], have been developed and applied in breast cancer research. These antagonists have shown inhibitory effects on tumor proliferation and invasion in preclinical models and can enhance the efficacy of PARP inhibitors, endocrine therapy, and radiotherapy [195–201]. Neutralizing antibodies against CXCL12 can also inhibit tumor growth [202]. Additionally, restoring the downregulated p16^INK4A^ expression in breast CAFs reduces CXCL12 secretion and inhibits the pro-tumorigenic function of CAFs [203], representing a potential therapeutic intervention strategy.

The IL-6/JAK/STAT3 pathway is aberrantly hyperactivated in many types of cancers and participates in the activation of CAFs, ultimately leading to poor clinical prognosis [204]. Targeting this pathway holds promise for therapeutic benefits. Tocilizumab, an IL-6 receptor antibody, can restore the normal phenotype of activated breast CAFs and inhibit their paracrine pro-tumorigenic effects [205]. It can also suppress the proliferation and migration of TNBC cells and enhance the cytotoxicity of cisplatin [206]. The JAK1/2 inhibitor ruxolitinib can inhibit the proliferation and migration of tamoxifen-resistant breast cancer cells [207] and has also shown antitumor activity in clinical trials [208]. In TNBC, targeting DCLK1 eliminates the malignant phenotype of cancer cells, enhances chemotherapy efficacy, and stimulates antitumor immunity by inhibiting the IL-6/STAT3 pathway [209].

The hepatocyte growth factor (HGF)/MET signaling pathway is also involved in the regulation of breast cancer by CAFs. HGF secreted by CAFs activates MET signaling, enhancing the colony-forming ability of cancer cells, and the use of HGF neutralizing antibodies can effectively reduce CAF-mediated breast cancer proliferation and invasion [210]. The MET inhibitor cabozantinib demonstrates therapeutic potential for TNBC with MET overexpression [211] and has shown certain efficacy in clinical trials for metastatic TNBC patients [212, 213].

Additionally, several other signaling pathways related to CAFs have also shown potential for targeted therapy. For example, the Notch signaling pathway mediates cancer migration and invasion in TNBC, while γ-secretase inhibitors (GSIs) such as DAPT can inhibit CAF-mediated enhancement of TNBC invasion by blocking Notch receptor activation [214]. GSIs also induce G2/M cell cycle arrest and trigger apoptosis in breast cancer cells, thereby inhibiting their survival [215]. Furthermore, in breast cancer, the ligand-dependent Hedgehog (Hh) signaling pathway not only serves as one of the key pathways for the transformation of NFs into CAFs [216] but also promotes the expansion and self-renewal of CSCs through its aberrant activation, and the Hh pathway inhibitor vismodegib can effectively block this effect [217], retard tumor growth [218], and enhance the penetration of chemotherapeutic drugs into the TME [219]. The Hh inhibitor sonidegib has also demonstrated promising efficacy in clinical trials for TNBC [220]. CAFs also promote the growth and invasion of breast cancer cells through a paracrine FGF2-FGFR1 loop [221], while FGFR blockade inhibits CAF functions and promotes T cell infiltration [222]. Moreover, the tyrosine kinase inhibitor dovitinib can significantly block CAF-induced breast cancer invasion by inhibiting PI3K/Akt/mTOR signaling [223]. Inhibiting lysyl oxidase-like 2 (LOXL2), which is associated with poor prognosis, can also reduce breast cancer invasion and ECM deposition [224, 225]. OPN secreted by tumor cells can reprogram NFs into pro-tumorigenic CAFs, and knockdown of OPN in tumor cells attenuates stromal activation and inhibits tumor growth [226]. In basal-like breast cancer, there is a paracrine crosstalk between cancer cells expressing PDGF-CC and CAFs. Blocking PDGF-CC converts basal-like breast cancer into a hormone receptor-positive phenotype, thereby conferring sensitivity to endocrine therapy [227]. CAFs can also induce chemotherapy resistance in TNBC cells by activating IFN signaling, and blocking IFN receptors can partially reverse this effect, providing a new approach to overcome chemotherapy resistance [228].

### Targeting CAF-derived ECM components

The dynamic complexity of the TME, especially the extensive remodeling of the ECM, is a key factor influencing breast cancer progression and treatment response, and CAFs are the primary contributors to this fibrotic ECM [229]. By secreting components such as collagen, HA, and Tenascin-C (TNC), CAFs significantly alter the physical properties of the TME [230]. Therefore, targeting CAF-derived ECM components has become one of the important strategies for improving breast cancer treatment outcomes.

The excessive deposition and cross-linking of collagen secreted by CAFs increases tumor tissue stiffness, which not only promotes tumor growth and invasion [229], but also collaborates with HA to compress tumor blood vessels, thereby reducing the effective delivery of therapeutic drugs [231]. In this regard, the angiotensin receptor blocker (ARB) losartan can significantly reduce the secretion of collagen and HA by CAFs through inhibiting TGF-β1-mediated pro-fibrotic signaling, thereby improving tumor vascular perfusion and drug delivery efficiency [231], enhancing the effects of chemotherapy, radiotherapy, and immunotherapy [189, 232–234], and significantly inhibiting breast cancer progression [235]. Another anti-fibrotic drug, pirfenidone (PFD), can also inhibit collagen synthesis by antagonizing TGF-β signaling, significantly suppressing the growth and metastasis of breast cancer cells when combined with chemotherapy [236]. Furthermore, LOX promotes collagen cross-linking, and targeting LOX restores chemosensitivity of TNBC, exerting a favorable antitumor effect [237, 238].

HA is another important ECM component synthesized by CAFs [239]. It drives tumor progression through multiple mechanisms, including forming physical barriers, activating CAFs, and promoting tumor cell invasion [240]. Studies have shown that the HA-associated physical barrier limits immune cell access to tumors, while the HA-degrading enzyme PEGPH20 significantly increases immune cell infiltration and enhances the efficacy of trastuzumab and anti-PD-L1 antibody [241, 242]. PEGPH20 also demonstrated certain efficacy in phase I clinical trials for advanced solid tumors [243]. However, in a phase III clinical trial for pancreatic cancer, PEGPH20 failed to improve overall survival [244], and its activity in combination with atezolizumab was relatively limited [245], indicating that its application potential in breast cancer needs further validation. Additionally, study has found that reduced HA cross-linking levels are associated with increased breast cancer malignancy, suggesting that recovering HA cross-linking may be a potential intervention strategy [239]. On the other hand, the development of antitumor drug carriers utilizing the properties of HA has demonstrated application potential in breast cancer treatment [246–248].

TNC promotes immunosuppression in breast cancer and is associated with enhanced tumor invasiveness and poor prognosis [249, 250]. In TNBC models, inhibition of TNC expression sensitizes T cell-mediated tumor killing and improves the antitumor effect of anti-PD-1/PD-L1 therapy [249]. F16-IL2 is a fusion protein targeting the A1 domain of TNC, which significantly enhanced the therapeutic efficacy when used in combination with chemotherapy in breast cancer models [251]. Furthermore, radioimmunotherapy targeting TNC has demonstrated therapeutic potential in brain tumors [252], providing a reference for exploring its application in breast cancer.

### Other targets for CAFs

In addition to the above targets, other biological characteristics of CAFs also provide new intervention directions for breast cancer treatment. For example, epigenetic dysregulation in CAFs may enhance breast cancer cell survival, and epigenetic modulators targeting histone modifications and microRNAs (miRNAs) provide new insights for breast cancer therapy targeting CAFs [253].

HDAC-mediated modifications are essential for maintaining CAF gene expression and function [253], while the HDAC inhibitor (HDACi) scriptaid can delay tumor growth by reversing CAF activation and function [254]. Vorinostat is a broad-spectrum HDACi that has shown reliable efficacy and safety in phase I clinical trial for metastatic breast cancer patients [255], and is capable of reversing hormone resistance in breast cancer [256]. Another HDACi, entinostat, also demonstrated antitumor activity in the treatment of patients with estrogen receptor-positive advanced breast cancer [257]. It is worth noting that some studies have found that fibroblasts treated with HDACi can instead promote tumor growth in vivo [258], indicating a dual nature of their effects.

Abnormal expression of multiple miRNAs in CAFs is also closely correlated with poor tumor prognosis [259]. As one of the most extensively studied pro-oncogenic miRNAs, miR-21 is typically highly expressed in CAFs [260] and is closely associated with lymph node metastasis in breast cancer [261]. Its small molecule inhibitor AC1MMYR2 inhibits tumor progression by reversing EMT [262], reprograms CAFs to suppress breast cancer lung metastasis [261], and counteracts paclitaxel-induced cancer metastasis [263]. Inhibition of miR-221 expression also abolishes CAF-induced breast cancer growth and migration [264]. Conversely, downregulation of miR-146b-5p drives CAF activation and promotes breast cancer invasion, while restoring its expression with curcumin effectively inhibits the pro-tumorigenic function of CAFs [253]. Furthermore, miR-9 secreted by tumor cells induces the transformation of NFs into CAFs via exosomes, and targeting miR-9 reverses the invasive phenotype of CAFs [265]. Conversely, miR-1-3p expression is downregulated in extracellular vesicles secreted by CAFs, while transfection with its mimic effectively inhibits breast cancer progression and metastasis, highlighting the therapeutic potential of miRNA targeting [266].

Collectively, the abundant stromal ECM components and CAFs within the breast cancer TME, along with their unique biological properties, have created opportunities for the development of multifaceted therapeutic strategies, which have emerged as critical directions for enhancing anticancer therapies (Table 3). Moving forward, in-depth research on the cellular heterogeneity and functional dynamics of CAF subpopulations is imperative, which will not only facilitate the development of precision medicine strategies but also create conditions for reducing off-target side effects and maintaining the homeostasis of normal stromal cells.

**Table 3:** Overview of CAF-associated therapeutic strategies in breast cancer

Target	Drug	Class	Mechanism	Combination Therapy	Preclinical/Clinical	Refs.
FAPα^+^ CAFs	CpVR-FAP	DNA Vaccine	Induce FAPα-specific CTL responses to directly kill FAPα^+^ CAFs	None	Preclinical	[157]
FAPα^+^ CAFs	Ad-FAP	Adenovirus Vector Vaccine	Induce FAP-specific CTL responses to directly kill FAP⁺ CAFs	DNA Vaccine, Cyclophosphamide	Preclinical	[163]
FAP^+^ CAFs	OMTX705	Antibody-Drug Conjugate	Specifically binds to CAFs, internalizes to release cytotoxin TAM470	PD-1 Inhibitor	Preclinical	[167]
FAP^+^ CAFs	αFAP-PE38	Immunotoxin	Induces apoptosis of FAP^+^ CAFs	Paclitaxel	Preclinical	[168]
α-SMA^+^ CAFs	NanoPue	Nanoemulsion	Downregulates ROS, reduces α-SMA⁺ CAFs and collagen deposition	NanoPTX, PD-L1 Inhibitor	Preclinical	[177]
PDPN^+^ CAFs	PDPN-IR700 Conjugate	Photoimmunotherapy	Reduce PDPN^+^ CAFs and enhance CD8^+^ T cell infiltration	None	Preclinical	[179]
CD10^+^GPR77^+^ CAFs	Anti-GPR77 Antibody	Monoclonal Antibody	Inhibit NF-κB pathway, reduce IL-6/IL-8 secretion, and disrupt the survival niche of CSCs	Docetaxel	Preclinical	[180]
TGF-βRI	IN-1130, YR-290	Small-Molecule Inhibitor	Inhibit the TGF-β/Smad signaling pathway	None	Preclinical	[183, 184]
TGF-βRI	Galunisertib	Small-Molecule Inhibitor	Inhibit the TGF-β/Smad signaling pathway	PD-L1 Inhibitor	Preclinical	[186]
TGF-β	Fresolimumab	Monoclonal Antibody	Neutralize TGFβ1/2/3	Focal Radiotherapy	Phase II	[188]
PD-L1, TGF-β	M7824	Bifunctional Fusion Protein	Block the PD-L1/PD-1 signaling and neutralize TGF-β1/2/3	Radiotherapy, Chemotherapy, Tumor Vaccine	Preclinical	[192]
CXCR4	LY2510924	Peptide Antagonist	Block the CXCR4/SDF-1 signaling pathway	None	Phase I	[195]
CXCR4	POL5551	Peptide Antagonist	Block the CXCR4/SDF-1 signaling pathway	Eribulin	Preclinical	[196]
CXCR4	AMD3100, AMD3465	Small-Molecule Inhibitor	Block the CXCR4/SDF-1 signaling pathway	None	Preclinical	[197, 198]
IL-6R	Tocilizumab	Monoclonal Antibody	Inhibit the activation of CAFs	None	Preclinical	[205]
JAK1/2	Ruxolitinib	Small-Molecule Inhibitor	Block the JAK2-STAT3 signaling pathway, reduce the survival and proliferation of breast CSCs	Paclitaxel	Phase I	[208]
MET	Cabozantinib	Small-Molecule Inhibitor	Inhibit the VEGFR/MET pathway to reduce angiogenesis	Nivolumab	Phase II	[212]
Hedgehog pathway	Vismodegib	Small-Molecule Inhibitor	Inhibit CAF proliferation and activation	Gemcitabine, Abraxane, Doxil	Preclinical	[219]
Hedgehog pathway	Sonidegib	Small-Molecule Inhibitor	Inhibit the Smo/Hedgehog signaling pathway to block its oncogenic effects	Docetaxel	Phase I	[220]
PI3K/Akt/mTOR	Dovitinib	Small-Molecule Inhibitor	Reduce the secretion of CCL2/CCL5/VEGF, inhibit breast cancer invasion	Ly294002, RAD001	Preclinical	[223]
Angiotensin II receptor	Losartan	Small-Molecule Inhibitor	Degrade the ECM and improve perfusion	Doxorubicin, PD-1 Inhibitor	Preclinical	[232]
TGF-β/SMAD	Pirfenidone	Small-Molecule Inhibitor	Reduce collagen secretion and decrease tumor stromal stiffness	Nab-PTX	Preclinical	[236]
Hyaluronan	PEGPH20	Hyaluronidase	Degrade hyaluronan and enhance tumor vascular perfusion	None	Phase I	[243]
HDAC	Scriptaid	Small-Molecule Inhibitor	Inhibit CAF differentiation	None	Preclinical	[254]
HDAC	Vorinostat	Small-Molecule Inhibitor	Increase histone lysine acetylation and induce tumor cell apoptosis	Tamoxifen	Phase II	[256]
HDAC	Entinostat	Small-Molecule Inhibitor	Increase protein lysine acetylation and reverse aromatase inhibitor resistance	Exemestane	Phase II	[257]
MiR-21	AC1MMYR2	Small-Molecule Inhibitor	Inhibit CAF activation	Taxol	Preclinical	[261]

## Conclusions

The high heterogeneity of CAFs in the TME presents both challenges and opportunities for developing more effective therapeutic strategies for breast cancer. The complex interactions among multiple components in the TME drive the phenotypic diversity of CAFs, aiding in the elucidation of the complexity of the tumor ecosystem. However, using surface markers alone to identify different CAF subpopulations has limitations, and their association with patient prognosis is complex and contradictory, suggesting that a hypothesis-free approach to first identify subpopulations and biomarkers may be more crucial. This approach has only recently become feasible with the application of scRNA-seq technology. Many researchers are dedicated to comprehensively characterizing CAFs and their heterogeneous subpopulations by integrating multi-omics data within a spatial context, providing insights into the spatial organization, cellular neighborhoods and interactions of CAFs, as well as their impacts on infiltrating immune cells and patient survival. These studies enhance the understanding of the diversity of mechanisms promoting tumor progression and immunoregulation within the TME.

It should be noted that current research on CAF heterogeneity is moving towards deeper integrative analysis, particularly emphasizing its spatial dimension and dynamic evolution. Recent studies have divided solid tumors into three regions: tumor body (TB), proximal invasive front (PIF), and distal invasive front (DIF), revealing that the high-density radial ECM fibers in the DIF act as “active signal input” to drive cancer cell invasion [267]. The latest study also introduces the concept of spatial partitioning into tumor bed, tumor margin, and distant region. By analyzing the proportions of each CAF subpopulations and T cells within these regions, it validated the spatial dependency between CAF subpopulations and T cell infiltration [3]. This suggests that when studying CAF heterogeneity, we should also consider their spatial arrangements and communication networks, and focus on whether CAF subpopulations exert heterogeneous roles in different spatial partitions, thereby deciphering the spatial dependency mechanism of the tripartite interaction among CAFs, ECM, and immune cells, providing a new perspective for precisely blocking the escape route of tumors. Furthermore, the dynamic transformations among different CAF subpopulations suggest that CAF subpopulations represent different cellular states rather than unchanging cell types, which prompts a shift in the research paradigm from “static classification” to “dynamic trajectory analysis”.

The central role of CAFs in regulating the immune landscape is manifested through their interactions with immune cells within the TME, shaping unique cellular neighborhoods. These CAF subpopulations, possessing unique transcriptional profiles and functional properties, form distinct spatial organizations with immune cells. For example, specific CAF subpopulations form functionally specialized ECT through spatial co-localization and interactions with distinct immune cells, capable of both establishing immuno-protective microenvironment and facilitating the formation of immunosuppressive networks, thus exerting multidimensional regulatory capacities within the TIME [5]. Pan-cancer analysis further reveals significant correlations between the four spatial CAF subtypes and the abundance, distribution, and state compositions of TILs. Their heterogeneous features are conserved across different histological types and pathological stages of tumor development, highlighting the functional diversity and context-dependent roles of CAFs in shaping the TME [3]. These findings reveal the heterogeneous functional landscape of CAFs and highlight that their multifunctionality in immunoregulation is based on interactions with neighboring immune cells. Understanding this heterogeneity is crucial for developing new therapeutic strategies. Future research should investigate the role of heterogeneous CAFs in treatment response and drug resistance, track their spatial dynamics and temporal evolutionary trajectories during therapies, and compare CAF profiles between patients with different treatment responses, striving to facilitate the clinical translation of research on CAF heterogeneity.

However, the specific crosstalk between CAFs and other components within the TME across different breast cancer subtypes and disease stages still requires further elucidation. Understanding the factors driving CAF heterogeneity is essential for proposing therapeutic approaches that target tumor activity. Spatial dynamics indicate that CAFs play differential roles in regulating the distribution and function of immune cells in their neighborhoods, making precise analysis of neighboring cellular organization a critical step in advancing CAF research. Looking ahead, leveraging more advanced machine learning or single-cell spatial omics platforms with higher coverage will create new opportunities for predicting the spatial distributions and functional properties of CAFs. These approaches will provide a stronger theoretical foundation for CAF-based clinical treatment strategies, further promoting the transition from “pan-stromal intervention” to “subpopulation-specific therapy”. All these efforts are dedicated to unraveling CAF heterogeneity, evolutionary trajectories, functional plasticity, and complex crosstalk with various components of the TME, thereby guiding the development of innovative therapeutic strategies in oncology.

## Data Availability

No datasets were generated or analysed during the current study.

## Abbreviations

TME: Tumor microenvironment
ECM: Extracellular matrix
CAFs: Cancer-associated fibroblasts
TIME: Tumor immune microenvironment
MDSCs: Myeloid-derived suppressor cells
scRNA-seq: Single-cell RNA sequencing
BMSCs: Bone marrow mesenchymal stem cells
OPN: Osteopontin
bFGF: Basic fibroblast growth factor
ASCs: Adipose-derived stem cells
CSCs: Cancer stem cells
EMT: Epithelial-mesenchymal transition
EndMT: Endothelial-mesenchymal transition
α-SMA: α-Smooth muscle actin
FAP: Fibroblast activation protein
FSP1: Fibroblast specific protein 1
PDGFR: Platelet-derived growth factor receptor
Cav-1: Caveolin-1
DCIS: Ductal carcinoma in situ
IDC: Invasive ductal carcinoma
OS: Overall survival
DFS: Disease-free survival
RFS: Recurrence-free survival
CNS: Central nervous system
TNBC: Triple-negative breast cancer
HGSOC: High-grade serous ovarian cancer
myCAFs: Myofibroblastic CAFs
iCAFs: Inflammatory CAFs
HNSCC: Head and neck squamous cell carcinoma
NSCLC: Non-small cell lung cancer
ECT: EcoCellTypes
TAM: Tumor-associated macrophages
Tstr: Stress response T cells
IMC: Imaging Mass Cytometry
MIBI: Multiplexed Ion Beam Imaging
MHC: Major histocompatibility complex
PVL: Perivascular-like
NFs: Normal fibroblasts
IBC: Invasive breast cancer
apCAFs: Antigen-presenting CAFs
PDAC: Pancreatic ductal adenocarcinoma
vCAFs: Vascular CAFs
mCAFs: Matrix CAFs
dCAFs: Developmental CAFs
CTLs: Cytotoxic T lymphocytes
FASL: Fas ligand
HDAC: Histone deacetylase
Tregs: Regulatory T cells
CM: Conditioned media
CAR-T: Chimeric antigen receptor T-cell
ICB: Immune checkpoint blockade
Chi3L1: Chitinase 3-like protein 1
HA: Hyaluronan
dPVL: Differentiated-PVL
ilCAFs: Interferon-licensed CAFs
Tex: Exhausted T cells
Tn: Naive T cells
Tfh: Follicular helper T cells
Tcm: Central memory T cells
LAMs: Lipid-associated macrophages
NK: Natural killer
ILC1s: Type 1 innate lymphoid cells
ADCC: Antibody-dependent cellular cytotoxicity
MCs: Mast cells
DCs: Dendritic cells
TILs: Tumor-infiltrating lymphocytes
pDCs: Plasmacytoid DCs
eNVs: Exosome-like nanovesicles
ADCs: Antibody-drug conjugates
TGF-βRI: TGF-β type I receptor
HGF: Hepatocyte growth factor
GSIs: γ-Secretase inhibitors
Hh: Hedgehog
LOXL2: Lysyl oxidase-like 2
TNC: Tenascin-C
ARB: Angiotensin receptor blocker
PFD: Pirfenidone
miRNAs: MicroRNAs
HDACi: HDAC inhibitor
TB: Tumor body
PIF: Proximal invasive front
DIF: Distal invasive front
